# Triple-Phase Multidetector Computed Tomography in Distinguishing Canine Hepatic Lesions

**DOI:** 10.3390/ani11010011

**Published:** 2020-12-23

**Authors:** Ladislav Stehlík, Morena Di Tommaso, Francesca Del Signore, Michaela Paninárová, Rossella Terragni, Tommaso Magni, Luisa Pontonutti, Andrea Carloni, Monica Alberti, Angela V. De Magistris, Massimo Vignoli

**Affiliations:** 1 Small Animal Clinic, Faculty of Veterinary Medicine, University of Veterinary and Pharmaceutical Sciences Brno, Palackého tř. 1946/1, 61242 Brno, Czech Republic; paninarovam@gmail.com; 2 Faculty of Veterinary Medicine, University of Teramo, Piano D’Accio, 64100 Teramo, Italy; mditommaso@unite.it (M.D.T.); mvignoli@unite.it (M.V.); 3 Veterinary Clinic Pet Care, via Marzabotto 1/2 M-N, 40133 Bologna, Italy; terragni.rossella@gmail.com (R.T.); tommy.magni@libero.it (T.M.); 4 Veterinary Clinic Gaspardis, via Gaspardis 2, Visco, 33040 Udine, Italy; luisa.pontonutti@gmail.com; 5 Veterinary Hospital “I Portoni Rossi”, via Roma 57/a, Zola Predosa, 40069 Bologna, Italy; andreacarloni91@gmail.com (A.C.); monica.alberti@portonirossi.it (M.A.); angdemag@gmail.com (A.V.D.M.)

**Keywords:** hepatopathy, attenuation value, liver neoplasia, hemangiosarcoma, hepatic inflammation

## Abstract

**Simple Summary:**

The goal of this study was to find any associations between the histologic diagnosis and the radiodensity of liver lesions. Thirty-one dogs with focal or multifocal liver lesions undergoing computed tomography examination were included in the study. Computed tomography examinations were performed before and after the application of a contrast medium, and postcontrast images were obtained in three different vascular phases; the arterial, portal, and delayed venous phases. A histological diagnosis was subsequently obtained for all of the dogs. From the results, no significant differences were identified between the benign and malignant liver lesions, nor between the individual histological types of lesions. The conclusion from this study is that triple-phase contrast-enhanced computed tomography cannot differentiate between benign and malignant liver lesions. Biopsy and further histological analysis are necessary.

**Abstract:**

The liver has a unique vascular supply, and triple-phase contrast-enhanced computed tomography examinations are being performed in order to characterize liver lesions. This study aimed to look for any associations between the attenuation values of liver lesions and their histological classification. The inclusion criteria for this retrospective study were focal or multifocal liver lesions and histological diagnosis. All of the dogs underwent pre-contrast and triple-phase postcontrast computed tomography (CT) examinations with identical timings of the postcontrast series. Thirty-one dogs were included in the study, and various benign and malignant pathologies were identified. The results did not identify any significant differences between the benign and malignant liver lesions, nor between the individual histological diagnoses. Inflammatory lesions were significantly different compared to the normal liver parenchyma, and significant hypoattenuation was found in the portal and delayed venous phases. Hemangiosarcomas were significantly hypoattenuating to the normal liver parenchyma in the pre-contrast and arterial phases, and also to all of the benign lesions in the arterial phase. The other pathologies showed variable attenuation patterns in the different postcontrast phases, and differentiation was not possible. On the basis of this study, triple-phase contrast-enhanced computed tomography cannot differentiate between benign and malignant liver lesions, and biopsy and further histological analysis are necessary.

## 1. Introduction

The evolution of multidetector computed tomography (MDCT) has allowed us to scan the entire patient in a short time, and to evaluate the organs in different vascular phases of contrast media distribution [1,2,3]. Triple-phase contrast-enhanced MDCT (TP-MDCT) is especially recommended for liver imaging because of the unique vascular supply of the liver. In a normal healthy liver, about 70–80% of the blood supply is provided by the portal vein, and the rest is provided by the hepatic artery [4]. The opposite is true for liver tumours, which are supplied mainly by the hepatic artery [5,6]. The same was also confirmed for metastatic carcinomas in the liver of humans [7]. The arterial blood supply is even stronger when tumours increase their size [8]. The results of an experimental study on mice with hepatic adenocarcinoma showed that the portal vein was responsible only for 4% of the blood supply [9]. Several papers dealing with multiphase MDCT of the liver were recently published in veterinary medicine [1,2,3,10]. One of those studies confirmed the greater accuracy of triple-phase MDCT compared to ultrasonography in the prediction of benign versus malignant masses [3]. Another study proposed some specific postcontrast features of hepatocellular carcinoma (HCC), nodular hyperplasia, and hepatic metastasis in dogs [2]. The same study also suggested that the vascular dynamics of HCC differ between dogs and humans [2].

The present study aimed to characterize liver lesions based on the attenuation values provided by the TP-MDCT, and to find possible associations with histological diagnosis.

## 2. Materials and Methods

For this multicentric and retrospective study, the archives of three different veterinary hospitals were reviewed in order to find dogs that had focal or multifocal liver lesions identified on TP-MDCT examination between April 2016 and February 2019. The patients were included if a concomitant final histopathological diagnosis of the liver lesions had been obtained within 30 days from the CT study.

The studies were all acquired on 16 slice MDCT machines (LightSpeed, GE HealthCare, Milwaukee, WI, USA; Optima 540, Waukesha, WI, USA; Toshiba Aquilion, Toshiba Medical Systems, Nasu, Japan) with animals in sternal recumbency in helical scan mode, and with a slice thickness of 1.25 or 2.5 mm, depending on the dog’s size, a rotation time of 0.5 s, a pitch of 1.3:1, and a standard/soft-tissue reconstruction algorithm. All of the dogs had pre- and triple-phase post-contrast CT examination available. An intravenous injection of 600 mg/kg of iodinated non-ionic contrast medium was delivered by a power injector (MCT Plus, Medrad, Indianola, IA, USA; CT-9000 ADV, Mallinckrodt–Covidien, Liebel-Flarsheim, USA; EnVision, Medrad, Indianola, IA, USA) at the rate of 2 mL/s. The arterial, portal, and delayed venous phases were performed at 10–15 s, 30–40 s, and 90 s from the beginning of the contrast medium’s injection, respectively. All of the images were retrospectively reviewed by one board-certified veterinary radiologist (MV) who was blinded to final histological diagnosis; circular regions of interest (ROIs) with an area of at least 2 cm² were placed in the normal hepatic parenchyma and the lesion. The ROI was placed to avoid major hepatic vessels and the central area of the lesion when hypoattenuating in the pre-contrast study, representing fluid or necrosis. For each ROI, the mean attenuation values (HU, Hounsfield Units) were recorded. For the DICOM data revision and analysis, the commercial software OsiriX (v.8.0.2, Pixmeo, Geneva, Switzerland) was used. The images were evaluated with a window width of 400 HU and a window level 40 HU. If the difference between the lesion and the normal liver parenchyma was more than +20 HU, the lesion was assessed as being hyperattenuating. If it was less than −20 HU, it was assessed as being hypoattenuating [1].

### Statistics

The statistical analyses were performed with the Real Statistics Resource Pack software (Release 6.3) in Microsoft Excel (version 16.26; Microsoft Corporation, Redmond, WA, USA). The two-sample T-test compared the attenuation values of the lesions in malignant and benign pathologies. The attenuation values of the lesion and normal liver parenchyma were compared in benign pathologies, malignant pathologies, and selected individual pathologies using a paired T-test. A one-factor ANOVA mutually compared the attenuation values of the lesion in selected individual pathologies. A two-sample T-test was used to compare the mean attenuation value of each malignant pathology against the mean attenuation value of all of the benign pathologies, and each benign pathology against all of the malignancies. The pathologies represented in this study with only one case were excluded from the comparison when the individual pathologies were tested. All of the statistical analyses were considered significant when *p* < 0.05. 

## 3. Results

Thirty-one dogs were included in the study. The mean age (±SD) was 11.3 ± 2.72 years (a range of 5 to 16 years). The mean weight (±SD) of the dogs was 23.3 ± 10.82 kg (a range of 5 to 43 kg). The group consisted of 7 (23%) intact females, 10 (32%) spayed females, 13 (42%) intact males, and 1 (3%) castrated male. There were 10 (32%) crossbreeds, 3 (10%) Labrador Retrievers, 2 (7%) Border Collies, and one each of the following breeds: Miniature Schnauzer, Beagle, Cocker Spaniel, Bernese Mountain dog, Italian Hound, English Bulldog, Doberman Pincher, Australian Shepherd dog, Dachshund, Pointer, Golden Retriever, German Shepherd, Toy Poodle, Alaskan Malamute, West Highland White Terrier, and Flat-Coated Retriever. 

The following types of pathologies were identified by histological examination: hepatocellular carcinoma (HCC; 8; 26%), hepatic degeneration (HD; 7; 23%), inflammation (5; 16%), hepatic adenoma (HA; 4; 13%), hemangiosarcoma (HSA; 4; 13%), nodular hyperplasia (1; 3%), cholangiocarcinoma (1; 3%), and histiocytic sarcoma (1; 3%). There were 17 (55%) benign and 14 (45%) malignant pathologies. The group of hepatic degeneration consisted of amyloidosis (3), lipidosis (3), and glycogen storage (1). The inflammatory group was created by eosinophilic (1), sterile neutrophilic (2), septic neutrophilic (1), and lymphoplasmacytic (1) inflammation.

No significant differences between the mean attenuation values of the malignant and benign liver lesions were found in the pre-contrast, arterial, portal, or delayed venous phases. 

In malignant pathologies, significant differences were found between the mean attenuation values of the lesion and the normal liver parenchyma in the pre-contrast (*p* = 0.0001), arterial (*p* = 0.003), portal (*p* = 0.0045), and delayed venous (*p* = 0.0276) phases. In the benign pathologies, significant differences in the mean attenuation values between the lesion and the normal liver parenchyma were found in the pre-contrast (*p* = 0.0181), portal (*p* = 0.0161), and delayed venous phases (*p* = 0.0003) (Table 1). 

No significant differences were found in the mean attenuation values of the lesions among the selected pathologies (HA, HCC, HD, HSA, inflammation). The mean attenuation values of the normal liver parenchyma and the lesion in the individual pathological processes are summarized in Table 2. Table 3 presents the difference of the attenuation values between the lesion and the normal liver parenchyma as hypo-, iso- or hyper-attenuating.

**Figure 1:**
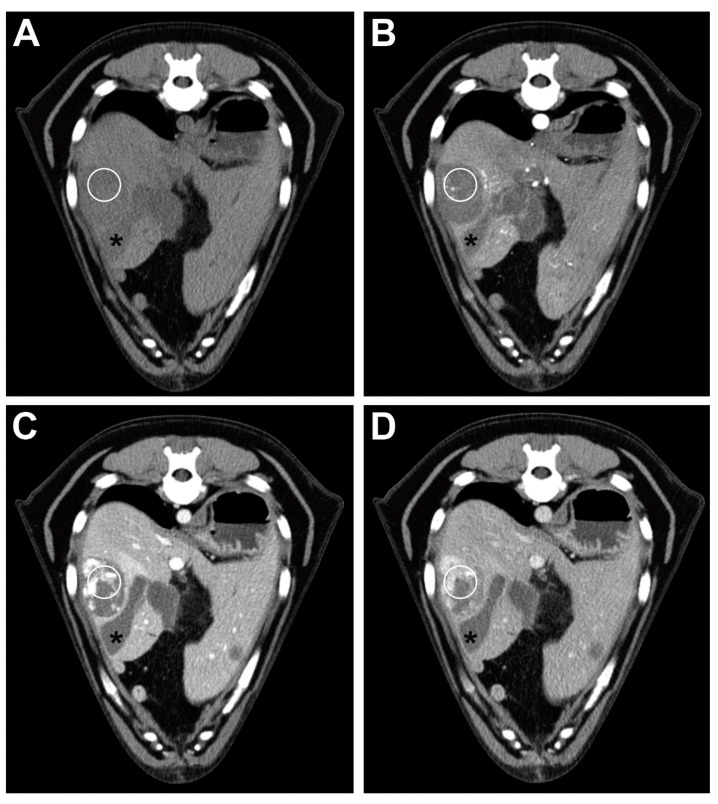
Transverse plane images of a TP-MDCT in a dog with hepatic hemangiosarcoma. The pre-contrast (**A**) and arterial (**B**) phase images show a hypoattenuating lesion in the right hepatic division. In the portal (**C**) and delayed (**D**) phase, the lesion is partially contrast-enhancing, with a non-enhanced hypoattenuating area. Asterisk = gallbladder; the white circle shows the location of the ROI used for the measurement.

All of the HSA cases were hypoattenuating in the pre-contrast and arterial phases. The portal and delayed phases did not show a unique attenuation pattern, and we observed one case with isoattenuating and hyperattenuating lesions in the portal and delayed phase, respectively (Figure 1). Another HSA case showed isoattenuation in the delayed phase. 

**Figure 2:**
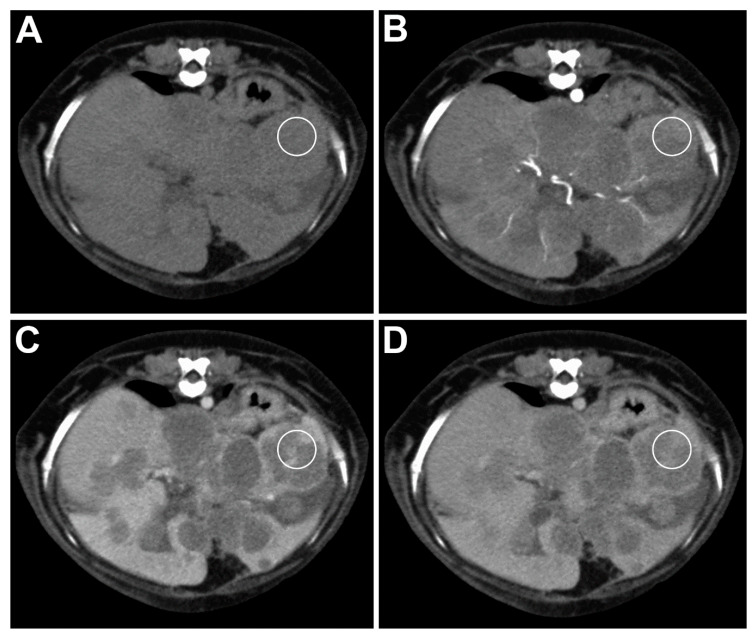
Transverse plane images of a TP-MDCT in a dog with HCC. (**A**) The lesions are isoattenuating in the pre-contrast phase. (**B**) Most of the lesions are isoattenuating in the arterial phase, and some are hypoattenuating. (**C**) The lesions are hypoattenuating in the portal phase. (**D**) In the delayed phase, some of the lesions are isoattenuating, and some are slightly hypoattenuating. The white circle shows the location of the ROI used for the measurement.

Most of the HCC lesions were isoattenuating compared to the normal liver parenchyma in all of the phases (Figure 2). Only one case presented as a hypoattenuating lesion in the pre-contrast and all of the postcontrast phases. 

**Figure 3:**
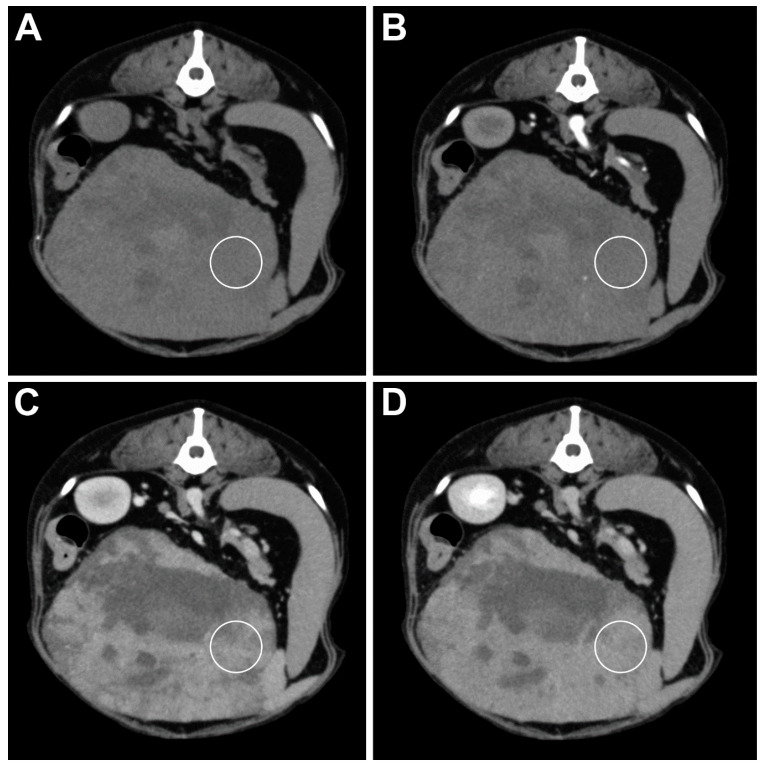
Transverse plane images of a TP-MDCT in a dog with a hepatic adenoma. (**A**) The pre-contrast image shows a large isoattenuating mass with multiple hypoattenuating areas. (**B**) In the arterial phase, the mass is of the same attenuation, and only a few tiny vessels are visible within the mass. The heterogenous contrast enhancement of the mass in the portal (**C**) and delayed (**D**) phase and the large hypoattenuating central area is present. The white circle shows the location of the ROI used for the measurement.

**Figure 4:**
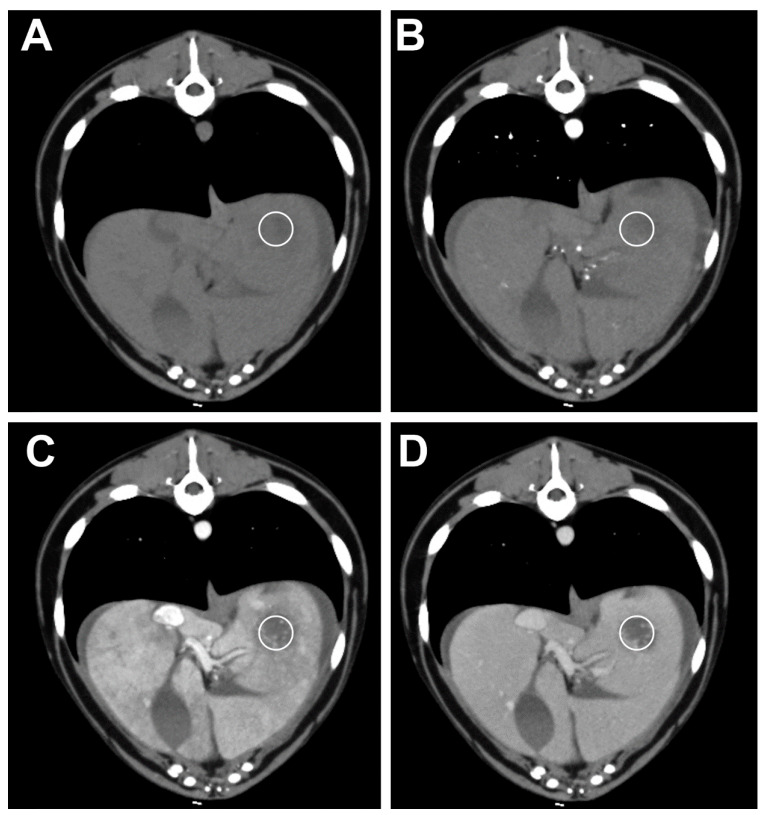
Transverse plane images of a TP-MDCT in a dog with hepatic inflammation. The pre-contrast (**A**) and arterial (**B**) phase images are of an isoattenuating lesion in the left hepatic division. In the portal (**C**) and delayed (**D**) phase images, the lesion is hypoattenuating with multiple tiny contrast-enhancing spots. The white circle shows the location of the ROI used for the measurement.

No significant differences were found between the mean attenuation value in the precontrast and all of the postcontrast phases of the HCC lesions and all of the benign lesions (*p* > 0.05). A significant difference was found between the mean attenuation value of the HSA lesion (46.5 ± 10.08 HU) and all of the benign lesions (65 ± 28.80 HU) in the arterial phase (*p* = 0.0484) (Figure 3 and Figure 4). 

There was a significant difference in the mean attenuation values in the delayed venous phase between the inflammatory lesions (57.4 ± 21.84 HU) and all of the malignant lesions (87 ± 30.23 HU). No significant differences were found in the mean attenuation values in the precontrast and all of the postcontrast phases of HD and HA compared to all of the malignant lesions.

## 4. Discussion

The results of our study did not confirm the possibility of distinguishing between benign and malignant liver lesions based on their attenuation values from TP-MDCT, compared to the previous studies [2,3,11]. However, significant differences were found between the attenuation values of the malignant lesion and the normal liver parenchyma in the pre-contrast and all of the post-contrast phases. The exact difference between the lesion and liver parenchyma was 15 HU on the pre-contrast and arterial phase images, and more than 20 HU on the portal and delayed venous phase images. In the case of benign pathologies, the lesions had significantly lower attenuation values compared to the normal liver parenchyma, except the arterial phase. The difference in the attenuation values in the arterial phase was approximately 5 HU. In the portal and delayed venous phases, the difference of the attenuation values was more than 20 HU, and in the pre-contrast images, it was 13 HU. Except for the arterial phase images, the differences in the attenuation values of the benign pathologies are very similar compared to the malignant lesions. It seems that the only possibility to distinguish malignant and benign pathologies is the arterial phase. However, no statistically significant difference was found between the benign and malignant lesions in the arterial phase. Considering these particular results from the practical point of view, there is no need to have a significant difference between the attenuation values. The difference in the attenuation value of the lesion and normal parenchyma needs to be big enough to be visible for the observer, and assessed as an hypo- or hyperattenuating lesion. However, there are no clear and unique guidelines for decision making regarding the hypo-, iso- or hyperattenuation of the liver lesion in veterinary medicine. A study about liver masses published in 2012 applied a difference of 20 HU between the lesion and normal liver parenchyma [1]. Later studies from 2014 and 2016 used, for this kind of lesion assessment, a difference of only 10 HU [2,10]. Based on our results, we can speculate that, in the arterial phase, the malignant lesion should be easier to assess as hypoattenuating (15 HU difference) compared to the benign lesion (5 HU difference). A recent study aimed at distinguishing benign and malignant liver lesions reported the usefulness of the delayed venous phase, in which the malignant lesions had a heterogeneous appearance [12].

Inflammatory lesions were significantly different to the normal liver parenchyma in all of the phases; however, no significant difference was found when they were compared to other liver pathologies. In the pre-contrast and arterial phases, the lesions were iso- or hypoattenuating. Except for one case, where the lesion was hypoattenuating in the pre-contrast and changed to isoattenuating in the arterial phase, the attenuation pattern in the remaining cases was unchanged between the pre-contrast and arterial phase. In the portal and delayed phases, the lesions were hypoattenuating in comparison to the normal liver parenchyma. Scientific peer-review literature describing the appearance of inflammatory liver changes in dogs on MDCT is not available. Sparse information about inflammatory liver pathologies could be found in the available veterinary CT textbooks. It is presented as diffuse changes causing enlargement and heterogeneous contrast enhancement [13,14]. A case report describing the CT features of lobular dissecting hepatitis presented hyperattenuating nodules in the pre-contrast and arterial phases, and isoattenuating nodules in the portal and delayed venous phases [15]. Such findings are different from our results, but lobular dissecting hepatitis was not present in our study group. The pre-contrast hyperattenuation of the liver nodules is not well understood, and various explanations have been offered. A role of dense collagen fibres between hepatocytes or copper accumulation in the liver have been hypothesized [15,16,17]. 

Degenerative hepatopathy in our study group presented with various attenuation patterns in different phases. The majority of cases were isoattenuating in the arterial and delayed phases. The information regarding this type of liver pathology is limited to a few reports dealing with steroid hepatopathy in dogs [18,19] and hepatic lipidosis in cats [20,21]. The attenuation values of the canine liver with steroid hepatopathy were measured only in the pre-contrast CT examination, and were reported as hyperattenuating [18,19]. Steroid hepatopathy is characterized by glycogen accumulation in the liver parenchyma [22]. Only one case of glycogen hepatopathy was confirmed in our study group, and the lesion was hypoattenuating compared to the normal liver parenchyma in the pre-contrast examination. 

Hepatic lipidosis was confirmed in three cases within our study group. In one case, a negative attenuation value of the lesion was observed in the pre-contrast examination. The lesions in the other two cases had attenuation values of 51 and 55 HU, respectively (normal liver parenchyma was 73 HU and 64 HU, respectively). There is only one case report available describing a negative liver attenuation value in a dog with hyperadrenocorticism where liver steatosis coexisted with glycogen liver dystrophy [23]. The authors reported the mean attenuation value of the liver as being −22.86 HU (±12.14 HU) in the pre-contrast examination and 22.93 HU (±17.84 HU) after the contrast application. Some guidelines for hepatic lipidosis on CT exist in human medicine. The attenuation value of the liver must be less than 48 HU [24], and the attenuation value of 40 HU means that approximately 30% of the parenchyma has fat infiltration [25].

Hepatic adenoma lesions showed various attenuations, and more often were hypo- or isoattenuating. None of them were hyperattenuating in the portal and delayed venous phases. A lack of information exists about this pathology in dogs. It was reported that hepatic adenomas are of variable contrast enhancement, and the majority are hypoattenuating in the arterial, portal and delayed phases [1]. It was also found that HA presents more often as a hyperattenuating lesion compared to HCC [1]. Hepatic adenoma in humans is mostly hypoattenuating in pre-contrast CT, hyperattenuating in the arterial phase, and hypoattenuating in the portal and delayed venous phases. However, it cannot be differentiated from the HCC and metastases [26]. 

In HCC, a significant difference between the lesion (50.9 HU) and normal liver parenchyma (62.0 HU) was found only in the pre-contrast examination. However, the mean attenuation values of the HCC lesions in our study in the arterial, portal, and delayed venous phases were lower than the normal liver parenchyma (a difference of 9 HU, 17 HU and 14 HU, respectively), but not significantly different. A study from human medicine reported that HCC nodules were hyperattenuating in the arterial phase, showing a wide range of attenuation profiles; however, they cannot be differentiated from regenerative or dysplastic nodules [27]. There are two studies on dogs reporting hyperattenuating HCC masses in the arterial postcontrast phase [2,28]. In another canine CT study, no HCC lesion was hyperattenuating in any of the postcontrast phases. In that study, HCC showed iso- (50%) or hypoattenuation (50%) in the arterial phase [1]. All of the canine studies found that the majority of HCC lesions to be of low density in the portal and delayed venous phases [1,2,28]. 

The HSA lesions in our study were hypoattenuating to the normal liver parenchyma in the pre-contrast and arterial phases, and these differences were found to be significant (a difference of 24 HU and 25 HU, respectively). In a previous study on dogs with hepatic masses, HSA was the major part of the metastatic masses (7 of 9), and all of the metastatic masses were hypoattenuating in the arterial phase and 88.9% in the portal phase [2]. The same study found 55.6% of metastatic masses to be hypoattenuating in the delayed phase, and the rest were isoattenuating [2]. The attenuation values of HSA lesions (46.5 HU) in the arterial phase were significantly different from all of the benign lesions (65 HU).

The limitations to our study are its retrospective nature, the small number of cases collected for each pathology, and the constant rate of contrast medium application. The same rate of contrast medium application (2 mL/s) and similar scanning time delays were used in a previously published study [10].

Another limitation could be the localization of the ROI. The measurements were taken at the same place within one patient in pre-contrast and all of the postcontrast phases. The size of the ROI was left unchanged within one patient. The size was not smaller than 2 cm^2^, but was larger if the lesion was huge and showed different degrees of postcontrast enhancement. The observer tried to encompass, in each case, all of the available densities within the lesion whilst avoiding the central and peripheral areas. The central area of the lesion was avoided because it mostly consists of necrotic tissue [1,9], and the peripheral parts were avoided due to the partial volume artefact. 

Furthermore, due to the multicentric retrospective nature of the study, the histologic diagnosis would have been made by various pathologists, although this is not considered to be a significant limitation. 

## 5. Conclusions

In our study, TP-MDCT did not allow us to differentiate between malignant and benign liver lesions, nor to differentiate among specific liver pathologies. The possibility to distinguish malignant and benign pathologies in the arterial phase needs further, larger, multicentric studies; however, the different enhancement of a hepatic lesion on the arterial phase could help to prioritize the tomographic differential diagnosis. The biopsy of liver lesions is still of paramount importance for definitive diagnosis. TP-MDCT could help to localize the lesion accurately, and to characterize the vascular supply of the liver lesions better and provide the critical information to plan the surgery. Future multicentric studies on a large group of dogs with variable liver pathologies using identical CT protocols are necessary.

## Figures and Tables

**Table 1:** Mean (±SD) attenuation values (HU) of the normal liver parenchyma and the lesion of the malignant and benign pathologies in the different phases of TP-MDCT.

Pathology	Pre-Contrast		Arterial		Portal		Delayed Venous	
	Normal	Lesion	Normal	Lesion	Normal	Lesion	Normal	Lesion
Malignant (*n* = 14)	63.1 (±9.02)	47.9 (±11.34) *	67.9 (±7.11)	52.8 (±17.24) *	113.8 (±11.43)	85.6 (±30.58) *	107.1 (±13.20)	87.0 (±30.23) *
Benign (*n* = 17)	60.9 (±8.69)	48.0 (±24.76) *	69.9 (±16.00)	65.0 (±28.80)	114.6 (±18.71)	90.5 (±42.41) *	112.8 (±7.09)	83.2 (±29.28) *

* Significantly different from the normal liver parenchyma; SD = standard deviation; HU = Hounsfield unit; TP-MDCT = triple-phase multidetector computed tomography.

**Table 2:** Mean (±SD) attenuation values (HU) of the normal liver parenchyma and the lesion in the different phases of TP-MDCT.

Pathology	Pre-Contrast		Arterial		Portal		Delayed Venous	
	Normal	Lesion	Normal	Lesion	Normal	Lesion	Normal	Lesion
Inflammation (*n* = 5)	59.2 (±7.89)	39.2 (±6.61) *	68.4 (±21.38)	48.2 (±14.48) *	121.4 (±23.42)	60.4 (±23.65) *	110.8 (±6.87)	57.4 (±21.84) *
Degenerative (*n* = 7)	59.7 (±10.48)	42.9 (±27.86)	71.3 (±18.46)	71.6 (±33.71)	114.7 (±14.44)	99.0 (±46.81)	117.4 (±4.89)	102.1 (±19.23)
HA (*n* = 4)	62.8 (±7.72)	67.3 (±31.16)	67.3 (±5.74)	67.0 (±31.55)	105.8 (±23.16)	95.8 (±33.03)	106.5 (±6.81)	73.5 (±26.41)
Nodular hyperplasia (*n* = 1)	70.0	51.0	78.0	95.0	115.0	161.0	115.0	119.0
HCC (*n* = 8)	62.0 (±8.78)	50.9 (±14.37) *	67.3 (±6.04)	58.5 (±20.40)	111.6 (±11.64)	94.9 (±19.33)	104.5 (±11.86)	90.5 (±26.15)
HSA (*n* = 4)	69.3 (±2.63)	44.8 (±4.27) *	71.8 (±9.60)	46.5 (±10.08) *	117.0 (±7.07)	69.0 (±49.44)	109.8 (±5.56)	87.0 (±43.36)
Cholangiocarcinoma (*n* = 1)	66.0	44.0	64.0	45.0	132.0	96.0	135.0	95.0
Histiocytic sarcoma (*n* = 1)	45.0	41.0	61.0	40.0	100.0	68.0	89.0	51.0

* Significantly different from the normal liver parenchyma; SD = standard deviation; HU = Hounsfield unit; TP-MDCT = triple-phase multidetector computed tomography; HA = hepatic adenoma; HCC = hepatocellular carcinoma; HSA = hemangiosarcoma.

**Table 3:** The frequency of different attenuation types in the particular pathologies in the different phases of TP-MDCT.

Pathology	Pre-Contrast			Arterial			Portal			Delayed Venous		
	Hypo	Iso	Hyper	Hypo	Iso	Hyper	Hypo	Iso	Hyper	Hypo	Iso	Hyper
Inflammation (*n* = 5)	3	2	0	2	3	0	5	0	0	5	0	0
Degenerative (*n* = 7)	3	4	0	0	6	1	4	2	1	1	6	0
HA (*n* = 4)	1	2	1	1	2	1	1	3	0	2	2	0
Nodular hyperplasia (*n* = 1)	0	1	0	0	1	0	0	0	1	0	1	0
HCC (*n* = 8)	1	7	0	2	5	1	1	7	0	1	7	0
HSA (*n* = 4)	4	0	0	4	0	0	3	1	0	2	1	1
Cholangiocarcinoma (*n* = 1)	1	0	0	0	1	0	1	0	0	1	0	0
Histiocytic sarcoma (*n* = 1)	0	1	0	1	0	0	1	0	0	1	0	0

TP-MDCT = triple-phase multidetector computed tomography; HA = hepatic adenoma; HCC = hepatocellular carcinoma; HSA = hemangiosarcoma.

## Data Availability

The data presented in this study are available on request from the corresponding author. The data are not publicly available due to privacy protection.
